# Associations between weight-adjusted waist index and fractures: a population-based study

**DOI:** 10.1186/s13018-023-03776-8

**Published:** 2023-04-10

**Authors:** Jianchun Tao, Yun Zhang, Caixia Tan, Wenfu Tan

**Affiliations:** Department of Traumatic and Pediatric Orthopedics, The Affiliated Second Hospital, Hengyang Medical School, University of South China, No. 30 Jiefang Road, Shigu District, Hengyang City, 421009 Hunan Province People’s Republic of China

**Keywords:** Weight-adjusted-waist, Fracture, Osteoporosis, BMI, NHANES, Obesity

## Abstract

**Introduction:**

The weight-adjusted waist circumference index (WWI) is a novel obesity evaluation indicator that appears to be superior to body mass index (BMI) and waist circumference (WC) in evaluating muscle and fat mass. The purpose of this study was to investigate the association between WWI and fractures among adults.

**Methods:**

In this cross-sectional study, multivariate logistic regression and smoothed curve fitting were used to investigate linear and nonlinear associations between WWI and fractures, based on data from 28,679 adult participants in the National Health and Nutrition Examination Survey (NHANES) from 1999 to 2018.

**Results:**

After adjusting for all covariates, the prevalence of hip/wrist/spine fractures among all participants was 1.09%, 8.87%, and 1.97%, respectively. A 1-unit increase in WWI was associated with a 5% increase in the odds of hip fractures [1.05 (1.01, 1.10)], and a 9% increase in the odds of spine fractures [1.09 (1.06, 1.13)], but not with the prevalence of wrist fractures [0.97 (0.94, 1.06)].

**Conclusions:**

Higher WWI was associated with an increased prevalence of hip fracture and spine fracture, but not wrist fracture.

## Introduction

Osteoporosis is a systemic skeletal disease marked by low bone mass and architectural degeneration, accompanied by increased bone fragility and fracture risk [1, 2]. In the United States alone, osteoporotic fractures currently account for more than 500,000 hospitalizations [3], and this number is increasing as the population ages [4, 5]. Therefore, early prevention of fractures and exploration of risk factors is critical [6], and advances in population-based studies have led to more accurate assessments of fracture risk and expanded the range of options available for fracture prevention [7, 8].

Obesity is a complex metabolic disease [9]. The prevalence of obesity has increased dramatically over the past few decades and is now at an unprecedented level: nearly one-third of the global population is obese [10, 11]. Although it has long been known that obesity may protect against osteoporosis and fractures [12–14], a significant amount of research has emerged in recent years that refutes this theory [15–19]. Body mass index (BMI) and waist circumference (WC), two commonly used obesity markers, fail to differentiate between muscle mass and fat mass [20], while body composition and body fat distribution have been proposed to more accurately reflect adverse metabolic characteristics [21, 22].

The weight-adjusted waist circumference index (WWI), originally proposed by Park et al. [23], was shown to be associated with age-related changes in body composition, such as loss of muscle mass and retention or gain of fat mass [24]. In addition, several studies have shown a significant positive association between WWI and cardiovascular disease [25–27], chronic kidney disease [28], and all-cause mortality [29].

However, no studies have assessed the association between WWI and fractures. As a result, we performed a cross-sectional study to investigate the relationship between WWI and fractures using data from the National Health and Nutrition Examination Survey (NHANES) 1999–2018.

## Methods

### Study population

**Fig. 1:**
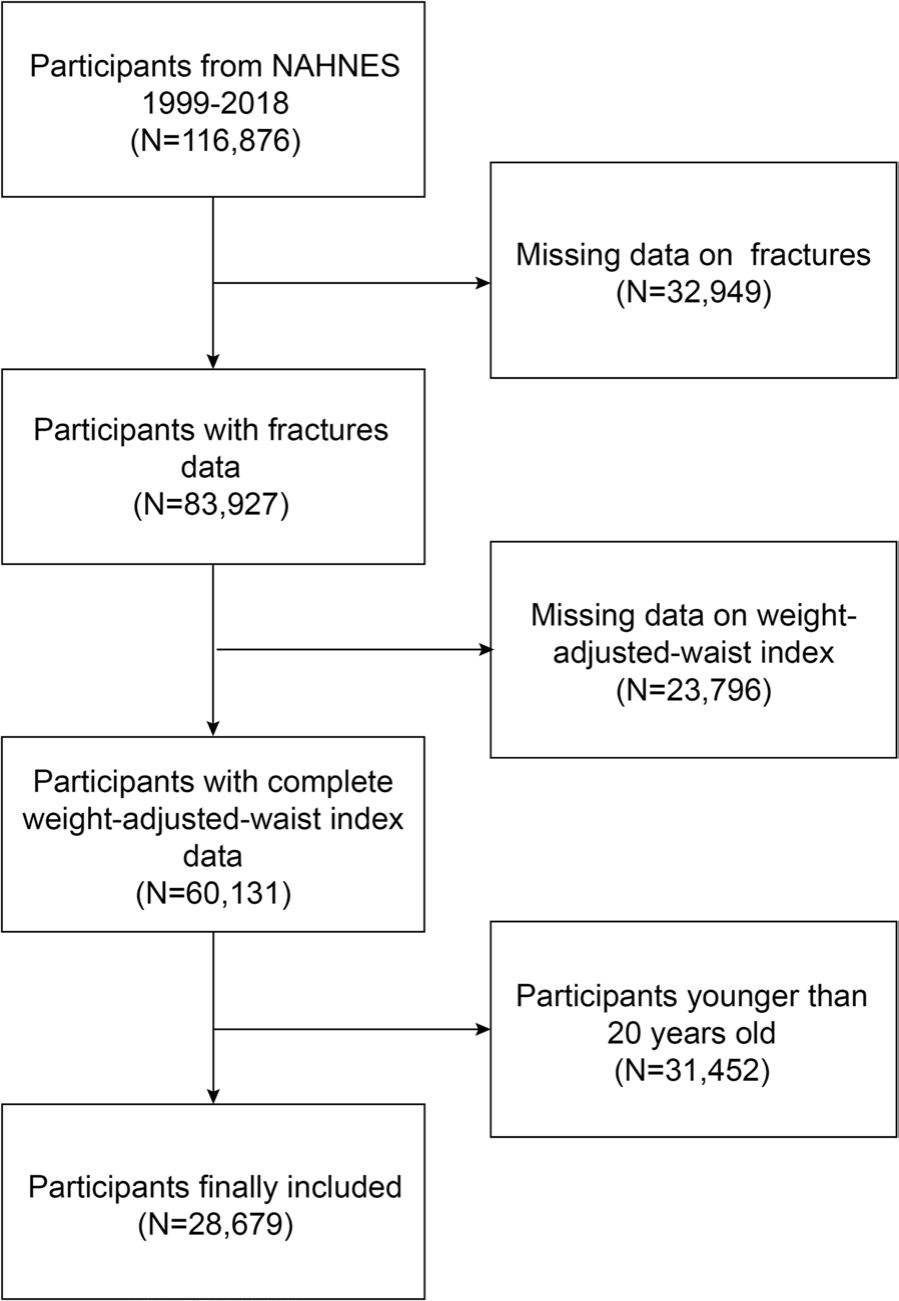
Flow chart of participants selection. NHANES, National Health and Nutrition Examination Survey

The NHANES, a program of the National Center for Health Statistics, is a well-known, nationally representative, cross-sectional survey that is performed across the United States [30, 31]. The National Center for Health Statistics (NCHS) Research Ethics Review Board approved the study procedure. At the time of recruitment, all participants provided written consent. The survey was conducted during 10 survey cycles over two decades (1999–2018). We excluded 32,949 participants without fractures questionnaire data, 23,796 participants with missing BMI or WC data, and 31,452 participants younger than 20 years old. The study eventually included 28,679 participants (Fig. 1).

### Study variables

WWI is an index to evaluate body fat mass and muscle mass, calculated by dividing WC (cm) by the square root of body weight (kg) [32]. At the mobile examination center, certified health technicians measured participants' weight and waist circumference. Participants' weights were determined by removing shoes and heavy clothing, and waist circumference was determined by drawing a horizontal line above the highest lateral border of the right iliac bone to plot the right mid-axillary line and placing a tape measure at the intersection of the two lines [33]. For the purpose of the participant's current fracture or fracture history diagnosis, participants were asked to recall whether they had previously been diagnosed with a hip/wrist/spine fracture by a professional orthopedic surgeon. An affirmative answer was identified as a current fracture or fracture history at the specific site [34, 35]. Covariates included demographic variables [age, sex, education levels, income-to-poverty ratio (PIR), and race]; examination variables [bone mineral density (BMD), waist circumference, and BMI]; laboratory variables [LDL-C (low-density lipoprotein cholesterol), total 25 (OH) D (25-hydroxy vitamin D), total calcium, and triglycerides]; dietary variables [dietary inflammatory index (calculated from 45 nutrient intakes)]; questionnaire variables [smoking status (Never/Ever/Current) [36], diabetes (Yes/No), use of hormone therapy (Yes/No)]. Comprehensive guidance regarding the collection methods of variables can be accessed from the NHANES Survey Methods (https://wwwn.cdc.gov/nchs/nhanes).

### Statistical analysis

As NHANES uses complex multi-stage sampling, we included appropriate weights in all statistical analyses in accordance with official guidelines [37, 38]. The study evaluated the characteristics of participants by dividing them into quartiles based on the WWI and employing chi-square and t-tests for analysis. Weighted multivariate logistic regression analysis was utilized to explore the linear relationship between WWI and fracture, and three models were developed to examine the association. Model 1 had no adjusted variables, Model 2 adjusted for age, gender, and race, and Model 3 adjusted for age, gender, race, smoking, dietary inflammatory index, diabetes, PIR, total 25 (OH) D, total calcium, use of hormone therapy, triglycerides, BMD, and LDL-C [39]. The association's strength was estimated using the odds ratio (OR) and its associated 95% confidence interval (CI) for the multivariate model. To assess the linear relationship between WWI and fracture, the researchers transformed WWI from a continuous variable to a categorical variable (quartiles) and analyzed trends using trend tests. Subgroup analyses were carried out to examine the association between WWI and fracture in individuals of varying gender, race, education, and diabetes status. Interaction tests were conducted to determine if the association was consistent across subgroups. For all results from multiple logistic regression analyses and subgroup analyses, we used the estimates from the fully adjusted model (Model 3) for interpretation. The researchers utilized smoothing curve fitting to explore the nonlinear relationship between WWI and fracture [40–43]. All analyses were performed using R (version 4.2) or Empowerstats (version 5.0), and a p-value < 0.05 was deemed statistically significant.

## Results

### Baseline characteristics

The mean (SD) age and mean WWI (SD) of the 28,679 participants were 49.99 (18.02) years and 11.02 (0.84), respectively. Of these, 48.01% were male and 51.36% were non-Hispanic white. The prevalence of hip/wrist/spine fractures was 1.09%, 8.87%, and 1.97%, respectively. Compared with the bottom WWI quartile, participants in the top WWI quartile were more likely to be female, Mexican American, and elderly; in terms of socioeconomic status, participants with higher WWI were more likely to have lower education and income; in terms of lifestyle, participants with higher WWI had higher rates of smoking and higher dietary inflammatory potential; in addition, participants with higher WWI typically had a higher prevalence of diabetes and fractures; had higher BMI, waist circumference, and lipid levels, and lower BMD, total 25 (OH) D, and total calcium (Table 1).

**Table 1:** Basic characteristics of participants by weight-adjusted-waist index quartile

Characteristics	Weight-adjusted-waist index				*P*-value
	Q1 (< 10.42) N = 7170	Q2 (10.42–11.01) N = 7169	Q3 (11.02–11.60) N = 7170	Q4 (> 11.60) N = 7170	
Age (years)	39.00 ± 14.61	47.31 ± 16.02	53.73 ± 16.82	59.94 ± 17.45	< 0.001
Sex, (%)					< 0.001
Male	52.84	52.01	45.61	36.15	
Female	47.16	47.99	54.39	63.85	
Race/ethnicity, (%)					< 0.001
Non-Hispanic White	52.86	45.25	48.55	47.60	
Non-Hispanic Black	26.51	17.05	15.92	12.28	
Mexican American	10.60	19.05	22.89	24.77	
Other race/multiracial	10.03	14.65	12.64	12.95	
Education level, (%)					< 0.001
Less than high school	12.04	15.39	20.37	25.83	
High school	20.21	23.48	24.90	26.10	
More than high school	67.75	61.13	54.73	48.07	
Use of hormone therapy, (%)					< 0.001
Yes	1.56	2.72	2.83	4.06	
No	98.44	97.28	97.17	95.94	
Smoking, (%)					< 0.001
Current	25.80	31.30	32.01	28.05	
Ever	41.99	46.44	48.90	50.10	
Never	58.01	53.56	51.10	49.90	
Diabetes, (%)					< 0.001
Yes	1.83	4.64	9.29	20.05	
No	98.17	95.36	90.71	79.95	
BMI (kg/m^2^)	24.56 ± 4.35	27.66 ± 5.01	29.79 ± 5.66	32.85 ± 7.18	< 0.001
Waist circumference (cm)	84.65 ± 10.07	95.45 ± 11.00	102.72 ± 12.00	112.42 ± 15.07	< 0.001
Total 25 (OH) D (nmol/l)	76.15 ± 1.37	75.16 ± 1.13	70.65 ± 1.18	64.65 ± 1.07	< 0.001
Total calcium (mmol/L)	2.45 ± 0.12	2.41 ± 0.09	2.37 ± 0.08	2.28 ± 0.09	< 0.001
PIR	3.18 ± 1.65	3.15 ± 1.63	2.98 ± 1.64	2.57 ± 1.58	< 0.001
DII	1.01 ± 1.86	1.27 ± 1.81	1.47 ± 1.78	1.74 ± 1.75	< 0.001
Triglycerides (mg./dL)	106.40 ± 92.04	131.69 ± 101.96	147.06 ± 138.60	157.97 ± 140.90	< 0.001
LDL-C (mg/dL)	110.34 ± 33.20	119.28 ± 34.51	120.29 ± 37.35	117.02 ± 36.57	< 0.001
Lumbar BMD (g/cm^2^)	1.07 ± 0.15	1.03 ± 0.14	1.01 ± 0.15	1.00 ± 0.16	< 0.001
Pelvis BMD (g/cm^2^)	1.28 ± 0.18	1.29 ± 0.17	1.27 ± 0.17	1.21 ± 0.17	< 0.001
Femoral neck BMD (g/cm^2^)	0.86 ± 0.15	0.83 ± 0.15	0.81 ± 0.14	0.78 ± 0.15	
Total BMD (g/cm^2^)	1.16 ± 0.11	1.14 ± 0.11	1.11 ± 0.11	1.08 ± 0.12	
Hip fractures					< 0.001
Yes	0.70	1.02	1.24	1.42	
No	99.30	98.98	98.76	98.58	
Wrist fractures					< 0.001
Yes	9.90	7.98	8.88	8.72	
No	90.10	92.02	91.12	91.28	
Spine fractures					< 0.001
Yes	1.62	1.70	2.11	2.44	
No	98.38	98.30	97.89	97.56	

Mean ± SD for continuous variables: the P value was calculated by the weighted linear regression model

(%) for categorical variables: the P value was calculated by the weighted chi-square test

*Q* quartile, *PIR* Ratio of family income to poverty, *BMI* body mass index, *LDL-C* low-density lipoprotein cholesterol, *DII* dietary inflammatory index, *BMD* bone mineral density, 25 (OH) D, 25-hydroxy vitamin D

### Association between WWI and fractures

**Fig. 2:**
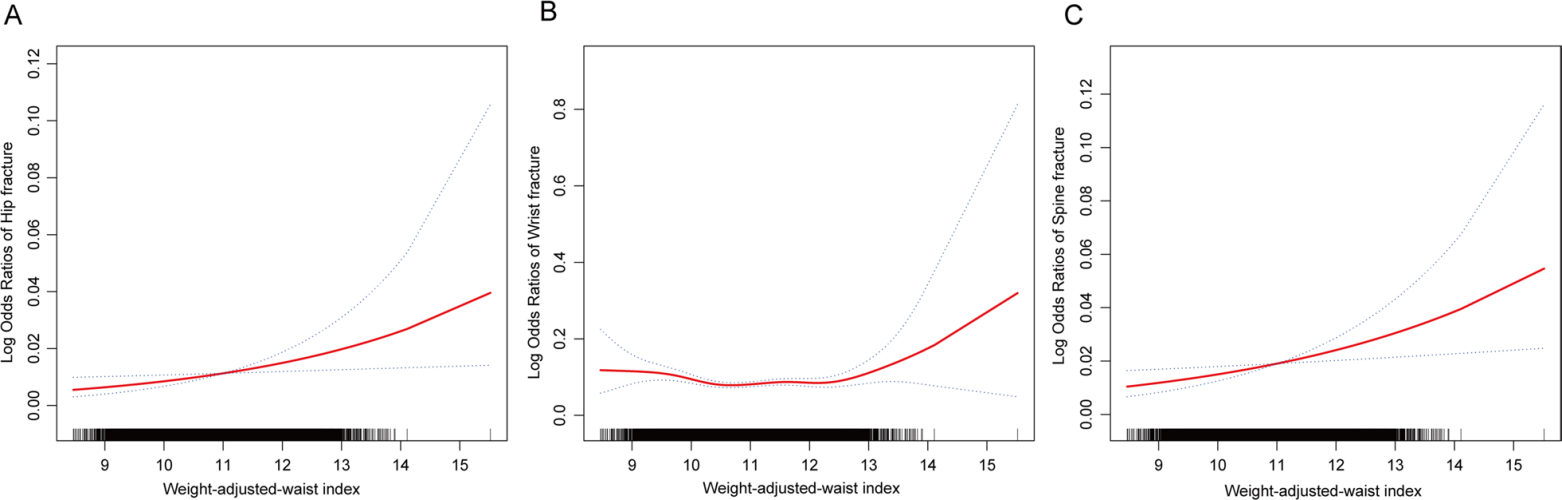
The nonlinear associations between weight-adjusted waist index and fractures. The solid red line represents the smooth curve fit between variables. Blue bands represent the 95% of confidence interval from the fit. **A**. WWI and hip fracture; **B** WWI and wrist fracture; **C** WWI and spine fracture. WWI, weight-adjusted waist index

Table 2 shows the associations between WWI and fractures. The results showed a significant positive linear association between WWI with hip fracture and spine fracture, while a non-significant negative association existed between WWI and wrist fracture. After adjusting for all covariates, each unit increase in WWI was associated with a 5% increase in the odds of hip fracture [1.05 (1.01, 1.10)] and an 9% increase in the odds of spine fracture [1.09 (1.06, 1.13)]. This significant positive association was maintained even when WWI was transformed into a categorical variable, with participants in the highest quartile of WWI having a 68% and 32% increased odds of hip fracture [1.68 (1.11, 2.01)] and spine fracture [1.32 (1.05, 1.55)], respectively. In addition, the results of the smoothed curve fitting further validated the nonlinear positive associations between WWI with hip fracture and spine fracture (Fig. 2).

**Table 2:** The associations between weight-adjusted-waist index and fractures

Exposure	Model 1 [OR (95% CI)]	Model 2 [OR (95% CI)]	Model 3 [OR (95% CI)]
Hip fractures (continuous)	1.30 (1.14, 1.50)	1.22 (1.04 1.40)	1.05 (1.01, 1.10)
Hip fractures (quartile)			
Quartile 1	Reference	Reference	Reference
Quartile 2	1.44 (1.00, 2.06)	1.32 (1.02, 1.80)	1.35 (0.95, 1.81)
Quartile 3	1.81 (1.28, 2.51)	1.33 (1.17, 1.62)	1.32 (1.03, 1.85)
Quartile 4	2.06 (1.47, 2.81)	1.59 (1.21, 1.97)	1.68 (1.11, 2.01)
P for trend	< 0.001	0.016	0.005
Wrist fractures (continuous)	0.96 (0.94, 0.98)	0.99 (0.94, 1.05)	0.97 (0.94, 1.06)
Wrist fractures (quartile)			
Quartile 1	Reference	Reference	Reference
Quartile 2	0.79 (0.70, 0.88)	0.79 (0.70, 0.89)	0.83 (0.72, 0.93)
Quartile 3	0.88 (0.79, 0.99)	0.93 (0.82, 1.05)	0.92 (0.77, 1.05)
Quartile 4	0.88 (0.77, 0.97)	0.91 (0.80, 1.04)	0.85 (0.78, 1.02)
P for trend	0.045	0.471	0.205
Spine fractures (continuous)	1.25 (1.15, 1.37)	1.17 (1.05, 1.30)	1.09 (1.06, 1.13)
Spine fractures (quartile)			
Quartile 1	Reference	Reference	Reference
Quartile 2	1.15 (0.89, 1.44)	0.99 (0.76, 1.29)	0.83 (0.64, 1.11)
Quartile 3	1.39 (1.03, 1.75)	1.11 (0.87, 1.47)	1.04 (0.76, 1.34)
Quartile 4	1.55 (1.23, 2.00)	1.35 (1.03, 1.67)	1.32 (1.05, 1.55)
P for trend	< 0.001	0.015	< 0.001

Model 1: no covariates were adjusted. Model 2: age, gender, race, and BMI were adjusted. Model 3: age, gender, race, smoking, dietary inflammatory index, diabetes, PIR, total 25 (OH) D, total calcium, use of hormone therapy, triglycerides, BMD, and LDL-C were adjusted

*PIR* Ratio of family income to poverty, *LDL-C* low-density lipoprotein cholesterol, *25 (OH) D* 25-hydroxy vitamin D

### Subgroup analyses

We conducted subgroup analysis and interaction tests stratified by age, gender, race, BMI, and diabetes to assess whether the relationship between WWI and fractures was consistent in the general population and identify any potential different population settings (Table 3). The results showed that the association between WWI and hip fracture was significantly different across the educational population, with participants below high school having a 39% and 65% higher prevalence of hip fracture than those in high school [0.60 (0.30, 1.02)] and above high school [0.36 (0.14, 1.02)], respectively. The association between WWI and fracture remained stable in the other subgroups (P for interaction > 0.05).

**Table 3:** Subgroup analysis of the association between weight-adjusted-waist index and fractures

Subgroup	Hip fractures [OR (95%CI)]	P for interaction	Wrist fractures [OR (95%CI)]	P for interaction	Spine fractures BMD [OR (95%CI)]	P for interaction
Sex		0.109		0.253		0.218
Male	Reference		Reference		Reference	
Female	1.05 (1.01, 1.10)		0.91 (0.85, 0.94)		0.94 (0.86, 1.02)	
Age		0.052		0.930		0.197
< 60 years	Reference		Reference		Reference	
≥ 60 years	0.65 (0.34, 0.98)		1.00 (0.85, 1.14)		0.84 (0.52, 1.18)	
Race/ethnicity		0.088		0.128		0.301
Non-Hispanic White	Reference		Reference		Reference	
Non-Hispanic Black	1.28 (0.73, 2.15)		0.75 (0.60, 1.02)		1.03 (0.54, 1.91)	
Mexican American	1.23 (0.74, 2.00)		0.79 (0.63, 1.04)		0.82 (0.53, 1.20)	
Other race	1.12 (0.71, 1.63)		1.09 (0.81, 1.40)		1.85 (1.10, 3.15)	
Education level		0.041		0.688		0.509
Less than high school	Reference		Reference		Reference	
High school	0.60 (0.30, 1.02)		0.96 (0.84, 1.16)		0.96 (0.71, 1.32)	
More than high school	0.36 (0.14, 1.02)		0.96 (0.81, 1.02)		1.08 (0.84, 1.40)	
Diabetes		0.852		0.601		0.811
Yes	reference		reference		reference	
No	0.89 (0.68, 1.15)		0.95 (0.87, 1.02)		1.06 (0.90, 1.25)	

Age, gender, race, smoking, dietary inflammatory index, diabetes, PIR, total 25 (OH) D, total calcium, use of hormone therapy, triglycerides, BMD, and LDL-C were adjusted

*PIR* Ratio of family income to poverty, *LDL-C* low-density lipoprotein cholesterol, *25 (OH) D* 25-hydroxy vitamin D

## Discussion

In the cross-sectional study that enrolled 28,679 eligible participants, we investigated the association between a new indicator of obesity, the WWI, and fractures at different sites. Our results suggest that elevated WWI is significantly associated with higher prevalence of hip fracture and spine fracture, but not wrist fracture. These findings suggest that WWI may be a valid indicator for assessing the association between obesity and fracture prevalence. The current findings underscore the significance of WWI in preventing and managing patients who are at a higher risk of experiencing fractures. Additionally, the findings provide a foundation for future research into the causal relationship between WWI and the prevalence of fractures.

To our knowledge, this is the first study to assess the relationship between WWI and fractures. In the past, obesity and being overweight have been considered a protective factor of osteoporosis and fractures. Several epidemiological studies with menopausal women have investigated the association between BMI and fracture risk, and these results suggest that an increase in BMI is associated with a decreased risk of fracture, with the most pronounced protective effect especially for hip fractures [18, 44–46]. However, studies contradicting these results have gradually increased. In a study that included 799 menopausal women, Premaor et al. found a significantly lower risk of wrist fracture and a significantly higher risk of hip fracture compared with women who were not obese [47]. The results of our large sample size study also suggest an association between rising WWI and higher hip fractures and a non-significant negative association with wrist fractures. A UK cohort study that included participants of different genders also showed that obese participants had a higher risk of ankle and upper arm fractures, but a 35% lower risk of wrist fractures [48]. The findings of epidemiological studies that contradict this long-held belief have spurred renewed interest in the paradigm shift regarding obesity as a protective factor for osteoporotic fractures [49].

On the one hand, with advances in investigation methods, several studies have identified nonlinear associations and saturation effects between BMI and fractures in a different ages, sex, and ethnic groups, and these results suggest that the association between BMI and fracture cannot be described simply by a linear positive correlation [15, 50, 51]. In addition, studies from different countries and regions have shown significant differences between BMI and fracture risk at different sites [47, 52, 53].

On the other hand, most studies investigating the association between obesity and fractures use BMI and WC to measure obesity and cannot distinguish between muscle mass, fat mass, and fat distribution. Gnudi et al. proposed a link between BMD with fat mass and muscle mass in women with osteoporosis, suggesting body composition is an essential element in research exploring bone metabolism [54]. The WWI is a unique anthropometric index that is considered to be a marker that can assess both high-fat mass and low-muscle mass [55]. The "obesity paradox" observed in the relationship between BMI or WC and metabolic diseases were observed to be less pronounced in WWI [56]. The current findings suggest that the obesity paradox may not exist but is attributable to BMI's inability to distinguish between muscle mass and fat mass [57]. The results of several recent epidemiological studies suggest that WWI outperforms BMI, WC, and waist-height ratios in the assessment of obesity and cardiovascular disease [58], sarcopenia [57], cardiac mortality, and all-cause mortality [23, 59].

The results of the subgroup analyses showed that the correlations between WWI and hip fractures differed among the subgroups of education level. Results similar to this finding have been reported in previous studies [60]. Results from a cross-sectional study investigating American men aged 20 years and older suggest that higher education and income are significantly associated with high lumbar BMD and that the educational attainment of participants should be fully considered in the prevention and treatment of osteoporotic fractures [61]. The data suggest that the effects of education on bone health are broad and complex and may affect bone metabolism in a variety of ways, including through income, cognition, occupation, and physical activity patterns [62–65]. Despite the consistency of the findings concerning significant differences between subgroups of educational attainment with prior research, caution is warranted in interpreting the significant outcomes observed in the subgroup analysis. There is a potential risk of false positives due to the failure to account for multiplicity between subgroups, and this possibility should be considered when interpreting the results.

The underlying mechanisms of this negative correlation between WWI and fractures are not fully understood. The metabolic characteristics of subcutaneous and visceral fat differ, and pro-inflammatory cytokines and tumor necrosis factor-alpha from visceral fat can accelerate bone resorption and so have a negative impact on bone metabolism [66]. Furthermore, there is compelling evidence that mesenchymal stromal/stem cells (MSC) are negatively associated with adipocytic and osteoblast commitment. The same mechanisms that govern MSC formation locally within the marrow microenvironment may act systemically between peripheral adipose depots and trabecular and cortical bone in cases of subcutaneous or visceral obesity [67, 68].

The strengths of our study include the use of a complex multi-stage probability sampling design and a large sample size, which increase the reliability and representativeness of our study. Our research has several limitations. First, we were unable to determine a causal association between WWI and fractures because to the design of the cross-sectional analysis. In addition, due to database limitations, we were unable to include data on all covariates that have an impact on bone metabolism, such as menopause, in order to maintain a sufficiently large sample size. Nevertheless, the current correlation between WWI and fractures was stable enough to be less likely to be significantly influenced by unincluded factors.

## Conclusion

Our results suggest that higher WWI is associated with an increased prevalence of hip fracture and spine fracture, but not wrist fracture. Further prospective studies and causal inference studies are needed to validate our findings.

## Data Availability

The survey data are publicly available on the internet for data users and researchers throughout the world ( www.cdc.gov/nchs/nhanes/).

## Abbreviations

WWI: Weight-adjusted waist circumference index
BMI: Body mass index
WC: Waist circumference
NHANES: National Health and Nutrition Examination Survey
NCHS: National Center for Health Statistics
LDL-C: Low-density lipoprotein cholesterol
Total 25 (OH): D (25-hydroxy vitamin D
PIR: Income-to-Poverty Ratio
MSC: Mesenchymal stromal/Stem cells
